# Sexual network characteristics, condomless anal intercourse, and the HIV care cascade among MSM living with controlled versus uncontrolled HIV infection in Lima, Peru: a population-based cross-sectional analysis

**DOI:** 10.1016/j.lana.2024.100722

**Published:** 2024-03-27

**Authors:** Carlyn L. Harris, Cherie S. Blair, Eddy R. Segura, Jessica Gutiérrez, Jordan E. Lake, Robinson Cabello, Jesse L. Clark

**Affiliations:** a Emory University School of Medicine, Atlanta, GA, USA; b South American Program in HIV Prevention Research, David Geffen School of Medicine at UCLA, Los Angeles, CA, USA; c Department of Medicine, David Geffen School of Medicine at UCLA, Division of Infectious Diseases, Los Angeles, CA, USA; d Facultad de Ciencias de la Salud, Universidad Peruana de Ciencias Aplicadas, Lima, Peru; e Asociación Civil Vía Libre, Lima, Peru; f UT Health Houston, Houston, TX, USA

**Keywords:** HIV prevention, Sexual networks, Men who have sex with men (MSM), Sexually transmitted infections (STIs), Detectable viremia

## Abstract

**Background:**

Despite high rates of HIV transmission among men who have sex with men (MSM) in Lima, Peru, limited data exist on the sexual network characteristics or risk factors for secondary HIV transmission among MSM with uncontrolled HIV infection. We report the frequency of serodiscordant, condomless anal intercourse (CAI) and associated sexual network characteristics among MSM in Lima with detectable HIV viremia and compare to those with undetectable viremia.

**Methods:**

This cross-sectional analysis includes MSM who tested positive for HIV-1 during screening for a trial of partner management and STI control (June 2022–January 2023). Participants were tested for HIV, gonorrhoea, chlamydia, and syphilis, and completed questionnaires on their demographic characteristics, sexual identity and behaviour, sexual network structures and engagement in HIV care.

**Findings:**

Of 665 MSM, 153 (23%) had detectable (>200 copies/mL) viremia. 75% (499/662) of men living with HIV were previously diagnosed, with 94% (n = 469/499) reporting that they were on ART, and 93% (n = 436/469) virally suppressed. 96% (n = 147/153) of men with detectable viremia reported serodiscordant CAI with at least one of their last three sexual partners, and 74% (n = 106/144) reported the same with all three of their recent partners. In contrast, 62% (n = 302/489) of men with undetectable viral load reported serodiscordant CAI with all of their last three partners (p < 0.01).

**Interpretation:**

23% of men living with HIV in Peru had detectable viremia, of whom almost all (96%) reported recent serodiscordant CAI. The primary gap in the HIV care cascade lies in awareness of HIV serostatus, suggesting that improved access to HIV testing could be a key prevention strategy in Peru.

**Funding:**

Funding for this study was provided by 10.13039/100000002NIH/10.13039/100000025NIMH grants R01 MH118973 (PI: Clark) and R25 MH087222 (PI: Clark).

**Research in context**

**Evidence before this study**

We searched PubMed for all articles relevant to sexual networks and condomless anal intercourse conducted in Peru. We used the search terms “HIV AND Peru AND (sexual networks OR condomless anal intercourse OR viral suppression)” with date restriction from 2000 to 2023 and no language restrictions. After title and abstract screen, 17 of 134 articles were relevant to our research question. While there was extensive evidence on sexual network dynamics of MSM in Peru, no studies specifically described the network characteristics and risk behaviour of MSM with detectable HIV-1 viremia.

**Added value of this study**

Our study is the first to describe the sexual network characteristics, frequency of serodiscordant condomless anal intercourse, and outcomes of the HIV care cascade among MSM living with HIV with detectable viremia and compares them to those with an undetectable viral load. Our findings reveal high rates of serodiscordant condomless anal intercourse among all participants, with higher frequencies reported among men with untreated HIV infection. These findings help explain the high incidence of HIV infection in Lima. In our population, the primary gap in the HIV care cascade lay in a lack of previous testing to identify men living with HIV and highlights a dire need to expand access to testing throughout Lima.

**Implications of all the available evidence**

Because of the high rates of serodiscordant condomless anal intercourse among MSM with detectable HIV viremia, it is important that individuals know their HIV status and are linked to care following an HIV diagnosis, to limit ongoing transmission. According to our results, among participants who knew their HIV status, 94% were taking ART and 93% were virally suppressed. Strategies to reduce barriers to testing and increase awareness of HIV status among MSM in Lima could significantly reduce secondary transmission of HIV. Based on previous literature, possible strategies include home self-testing, remote testing in sex-on-premises venues or bars or expanded access hours in traditional clinics.

## Introduction

In Lima, Peru, the HIV epidemic is concentrated among men who have sex with men (MSM) and transgender women (TW).^1^ The prevalence of HIV among MSM is estimated to be over 10%, and greater than 20% among TW, compared to roughly 0.3% in the general population.^2^ Previous studies have highlighted the importance of sexual network dynamics on secondary transmission of HIV in these populations.^3^, ^4^, ^5^, ^6^ However, no recent data exists on the individual and sexual network characteristics of MSM in Peru with detectable HIV viremia and their potential impact on HIV transmission. Because undetectable viremia essentially reduces an individual’s risk of HIV transmission to zero,^7^ interventions targeting persons with a detectable viral load have important implications for population-level HIV transmission dynamics.

Numerous studies have assessed factors influencing HIV transmission in Peru. For example, one study found that a large proportion of new infections stemmed from partners who were either unaware of their HIV diagnosis or aware but untreated.^3^ In another analysis, MSM living with HIV in Lima frequently reported condomless anal intercourse (CAI) with partners believed to be either HIV-negative or with unknown HIV serostatus, though this study did not assess for associations between condomless sex and detectable viral load.^8^ Only one study evaluated individual-level characteristics associated with detectable viremia among people living with HIV in Lima, finding that incomplete viral suppression was associated with transportation barriers to clinic appointments and diagnosis of an alcohol use disorder.^9^ However, this study did not assess the relationship between detectable viremia and sexual network characteristics and other risk factors for secondary transmission, such as number of partners, partnership types, partner serostatus, or frequency of condomless sex.

We report data on individual-level characteristics, sexual network structure, engagement in the HIV care cascade, and frequency of serodiscordant CAI in a sample of MSM living with uncontrolled HIV in Lima. We hypothesized that men with detectable HIV viremia would have distinct sexual network characteristics that sustain ongoing HIV transmission in the population and that this data could provide a guidepost for future HIV prevention and treatment interventions in the region.

## Methods

### Setting, participants, and recruitment

This cross-sectional analysis included MSM screened for a trial of Expedited Partner Therapy for Sexually Transmitted Infection (STI) control in Lima, Peru between June 2022 and January 2023. The study was performed at Via Libre, a non-governmental organization that provides HIV and STI care and education in communities throughout Lima. Research staff recruited participants at venues frequented by MSM or via referral through community allies. Inclusion was limited to individuals who (1) were at least 18 years old, (2) were assigned male sex at birth, and (3) reported condomless anal sex with a male or TW partner in the preceding 6 months. The inclusion criteria of a recent report of condomless anal intercourse were to capture participants at higher risk of sexually transmitted infections.

### Study procedures

All participants completed a computer-assisted survey (CASI) addressing participant- and partnership-level variables. Participant-level variables included demographic data (age and education), sexual identity, sexual role (*active* [insertive], *pasivo* [receptive], *moderno/versatile* [versatile]), number of sexual partners in the past three months, and the proportions of primary, casual, anonymous, and commercial partners within their recent sexual contacts. Participants were also asked about characteristics of their last three sexual contacts, including each partner’s gender, sexual identity, sexual role, age, and HIV status (if known), the type of partnership (stable, casual, anonymous, commercial), and whether the partner was taking HIV pre-exposure prophylaxis (PrEP) or antiretroviral therapy (ART). The research team also administered a sexual network interview that explored each participant’s number of recent sexual partners in the past 3 months, types of sexual partnerships, including how many partners were one-time versus repeated encounters, and frequency of CAI.

All participants were tested for HIV, including those who reported they were living with HIV. HIV-1 RNA was measured by PCR from a venous blood sample (Aptima HIV-1 Quant Dx; Hologic, San Diego, CA) for all participants who tested positive on screening. Only MSM living with HIV were included in this analysis.

### Statistical analysis

Medians with inter-quartile ranges ([IQR]) were reported for individual-level characteristics. Descriptive characteristics were compared between participants with detectable versus undetectable viremia using t-tests, Mann-Whitney-U, chi-square, and Fisher’s exact tests, as appropriate. “Detectable viremia” was defined as an HIV-1 viral load of 200 copies/mL or higher. We used this cut-off as previous studies have shown that chances of transmitting HIV are nearly zero when an individual’s viral load is below 200 copies/mL.^7^ To determine the frequency of serodiscordant CAI in this population, we defined a serodiscordant partner as someone the participant described as either seronegative or of unknown HIV status and defined recent CAI as receptive or insertive anal intercourse without a condom in the last 90 days. Analyses of serodiscordant CAI included the last three sexual partners identified by the participant. Frequency of serodiscordant CAI was compared between participants with detectable versus undetectable viremia using chi-square tests. Separate comparisons using chi-square tests were also conducted for participants who were newly diagnosed with HIV, and those who were already known to be living with HIV. Sub-analyses were also completed to examine insertive and receptive CAI separately.

Descriptive statistics for characteristics of the last three sexual partners are reported with frequencies for individuals with both detectable and undetectable viremia.

Engagement in the HIV care cascade was evaluated using the results of the HIV screening test, answers from the computer-assisted survey, and HIV viral load quantification. Participants who reported that they had never taken an HIV test, that their last HIV test was negative or indeterminate, or that they never received the result of their last HIV test were considered “Not Aware” of their diagnosis. Participants who reported that the result of their last HIV test was positive were considered “Aware” of their diagnosis. Participants who already knew they were living with HIV were asked to report if they were currently on ART and, if so, whether their viral load was undetectable at their last lab measurement.

We also conducted a sensitivity analysis of the above using a viral load cut-off of 1000 copies/mL instead of 200 copies/mL to confirm the robustness of our results.

14 participants were excluded due to missing viral load measurements. Data analysis was completed in STATA (StataCorp, LLC, Version 14).

### Human subjects’ protection

The University of California, Los Angeles Institutional Review Board and the Ethics Committee at *Asociación Civil Vía Libre* approved all study procedures before their implementation. All participants provided written informed consent prior to the initiation of any study activities or procedures including data and biological samples collection. The study from which this secondary analysis is derived was registered on ClinicalTrials.gov (NCT04553211).

### Role of the funding source

Funding for this study was provided by NIH/NIMH grants R01 MH118973 (PI: Clark) and R25 MH087222 (PI: Clark). The study sponsor did not have any involvement in the study design, collection, analysis, or the writing of this manuscript.

## Results

### Sample characteristics

**Fig. 1:**
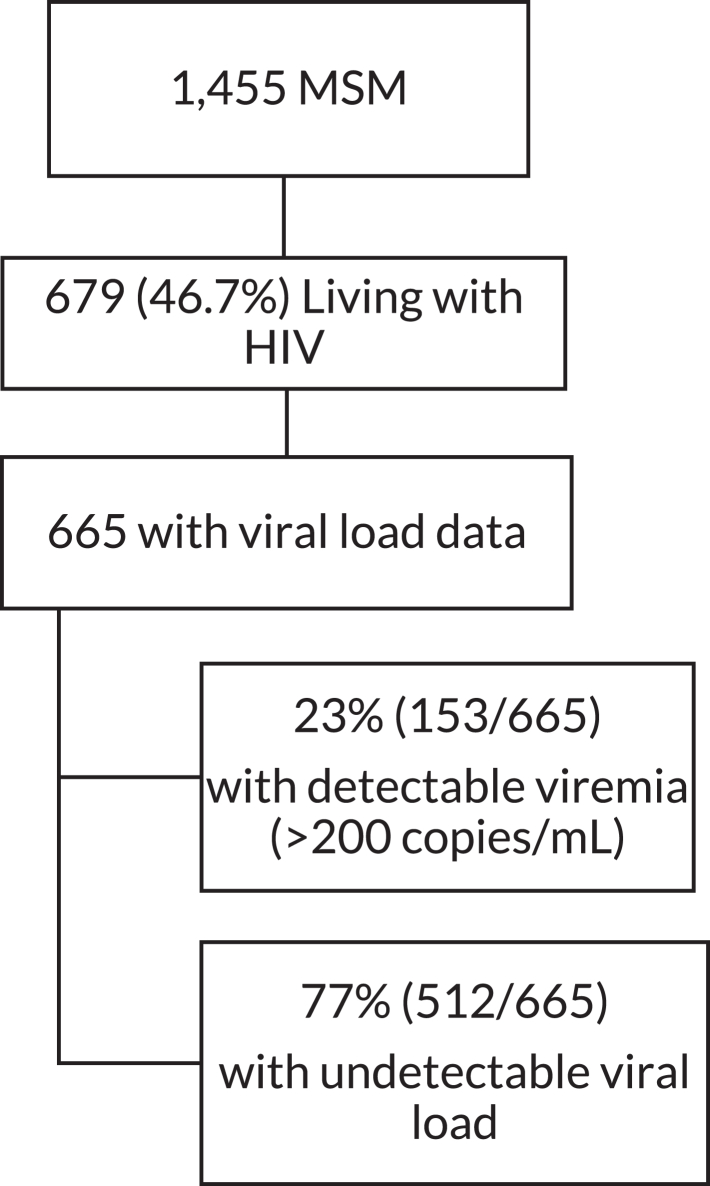
Study population of MSM living with HIV.

In our sample of 1455 MSM, 679 (46.7%) were living with HIV (Fig. 1). The median (IQR) age was 33 (27–35) with a range of 18–65. 44% of participants had a high school education or lower, 70% identified as homosexual, and 57% identified their sexual role during intercourse as “*moderno*” or “*versatil*” (Table 1). Among 665 MSM with available viral load data, 153 (23%) had detectable viremia, with a median viral load of 70,706 (IQR 9275–246,988) copies/mL. The median age of men with detectable viremia was 29 (IQR 25–35) years. Participants with detectable viremia were often younger (p < 0.001) and had completed less education (p < 0.001) than those with undetectable viremia. The median number of sexual partners in the last 3 months reported by men with detectable viremia was 15 (IQR 5–20), compared with 14 (IQR 6–25) among men with an undetectable viral load.

**Table 1:** Participant demographic and sexual network characteristics among MSM in Lima, Peru stratified by detectable versus undetectable viremia; 2022–2023.

Participant characteristics	Detectable HIV-1 viremia n (%)	Undetectable HIV-1 viremia n (%)
Age^a^	29 (25–35)^c^	34 (28–40.5)
Education		
High school graduate or below	67/153 (43.8)^c^	131/512 (25.6)
Technical or University	86/153 (56.2)	381/512 (74.4)
Sexual identity		
Heterosexual	3/153 (2.0)	7/509 (1.4)
Bisexual	39/153 (25.5)	97/509 (19.1)
Homosexual	107/153 (70.0)	389/509 (76.4)
Sexual role		
Active	18/153 (11.8)	62/512 (12)
Passive	44/153 (28.8)	134/512 (26.2)
Modern	87/153 (56.9)	310/512 (60.6)
Number of sexual partners in last 3 months^a^	15 (5–20)	14 (6–25)
Principal partners^a^	0 (0–1)	0 (0–1)
Casual partners^a^	13 (5–20)	13 (6–24)
Single contact casual partners^a^	8 (2–16)^b^	10 (3–19)
Single contact partners with insertive CAI^a^	2 (0–6)	2 (0–8)
Single contact partners with receptive CAI^a^	2 (0–8)	3 (0–10)
Repeat contact casual partners^a^	2 (0–5)	3 (1–5)
Repeat contact partners with insertive CAI^a^	1 (0–4)	1 (0–3)
Repeat contact partners with receptive CAI^a^	2 (1–4)	2 (1–4)
Anonymous partners^a^	5 (0–13)	6 (0–15)

CAI = Condomless Anal Intercourse; ART = Antiretroviral Therapy.

a Median (Interquartile range).

b p < 0.05.

c p < 0.001.

### HIV care cascade

Of the 665 MSM living with HIV with available viral load data, 3 left the question about previous HIV testing blank and were therefore left out of the following HIV care cascade analysis. 75% (n = 499/662) were aware of their HIV-positive status while 163/662 were newly diagnosed in this study, 94% (n = 469/499) of those who were aware of their HIV-positive status reported they were on ART, and 93% (n = 436/469) of men on ART were virally suppressed. 35.6% (n = 55/153) of participants with detectable viremia were aware of their HIV diagnosis, with 60% (n = 33/55) on ART and 45% (n = 15/33) stating that their viral load was undetectable at their last clinic visit. Of those who did not know they were living with HIV at the time of screening, 6% (n = 6/101) reported ever having been on PrEP and 1% (n = 1/101) were taking PrEP at the time of diagnosis. Participants who identified as homosexual were more likely to be aware of their HIV status (79%: n = 391/493), compared to those who identified as bisexual (60%: 82/136, p < 0.001). Participants who were unaware of their HIV status were also more likely to be younger (p < 0.001) and to have lower educational attainment (p < 0.01) compared to those who were previously aware. There was no difference in sexual role between those who were aware and unaware of their HIV status.

### Serodiscordant condomless anal intercourse

Most MSM living with HIV reported serodiscordant CAI with at least one of their last three sexual partners, including 96% of men with detectable viremia and 94% of those with undetectable viremia (Table 2). 74% of men with detectable viremia reported serodiscordant CAI with all of their last three partners, as compared to 62% (p < 0.01) of men with undetectable viremia. The frequency of serodiscordant receptive CAI with all three recent partners was 58% for participants with detectable viremia and 47% for participants with undetectable viremia (p < 0.05). The frequency of serodiscordant insertive CAI with all three partners was lower, and not significantly different between men with detectable (30%) and undetectable (26%) viral loads.

**Table 2:** Frequency of serodiscordant condomless anal intercourse (CAI) with the last three sexual partners, stratified by detectable and undetectable HIV viremia among MSM in Lima, Peru; 2022–2023.

Reported Serodiscordant CAI	Detectable viremia n/N (%)	Undetectable viremia n/N (%)
Serodiscordant CAI with any recent partner	147/153 (96.1)	477/510 (93.5)
Serodiscordant CAI with all 3 recent partners	106/144 (73.6)^b^	302/489 (61.8)
Serodiscordant insertive CAI with any recent partner	96/153 (62.8)	321/512 (62.7)
Serodiscordant insertive CAI with all 3 partners	43/144 (29.9)	129/489 (26.4)
Serodiscordant receptive CAI with any partner	131/153 (85.6)	421/512 (82.2)
Serodiscordant receptive CAI with all 3 partners	83/144 (57.6)^a^	232/489 (47.4)

Note: Denominators may vary due to some participants reporting less than 3 partners and missing data.

a p < 0.05.

b p < 0.01.

Differences in the frequency of serodiscordant CAI with recent partners were also noted among those who were newly diagnosed with HIV, with 98% reporting serodiscordant CAI with any of their last three partners and 76.5% with all of them, compared with 93.0% (p < 0.05) and 60.9% (p < 0.001), respectively, of those with a previous HIV diagnosis (Table 3). Serodiscordant insertive CAI with all three recent partners was reported more often by men with undiagnosed infection (40%) than by men who knew they were living with HIV (24%; p < 0.001).

**Table 3:** Frequency of serodiscordant CAI with the last three sexual partners, stratified by new HIV diagnosis and previous HIV diagnosis among MSM in Lima, Peru; 2022–2023.

	New HIV diagnosis n/N (%)	Previous HIV diagnosis n/N (%)
Serodiscordant CAI with any recent partner	159/163 (97.6)^a^	462/497 (93.0)
Serodiscordant CAI with all 3 recent partners	114/149 (76.5)^c^	290/481 (60.9)
Serodiscordant insertive CAI with any recent partner	115/163 (70.6)^a^	301/499 (60.3)
Serodiscordant insertive CAI with all 3 partners	59/149 (39.6)^c^	113/481 (23.5)
Serodiscordant receptive CAI with any partner	140/163 (85.9)	409/499 (82.0)
Serodiscordant receptive CAI with all 3 partners	89/149 (59.7)^b^	225/481 (46.8)

Note: Denominators may vary due to some participants reporting less than 3 partners and missing data.

a p < 0.05.

b p < 0.01.

c p < 0.001.

### Network characteristics

Participants with detectable viremia provided information about 447 partners in the previous 3-month period. All 153 participants reported data on at least one partner, 150 reported on two or more partners, and 144 reported on three recent partners.

Participants stated that 96% (n = 445) of their recent partners were male, with 61% (n = 446) described as homosexual, 28% as bisexual, and 7% as heterosexual. 43% of these sexual contacts were repeat casual partners and 42% were single-contact casual partners. Participants with detectable viremia did not know the HIV status of 74% of their most recent partners (Table 4).

**Table 4:** Characteristics of last three reported sexual partners among participants with detectable and undetectable viremia.

Partner Characteristics	Detectable HIV n (%)	Undetectable HIV n (%)
Gender		
Male/masculine	427/445 (96.0)	1482/1501 (98.7)
Woman/feminine	7/445 (1.8)	7/1501 (0.47)
Trans woman	4/445 (0.9)	9/1501 (0.6)
Trans man	7/445 (1.6)	3/1501 (0.2)
Sexual identity		
Homosexual	272/446 (61)	1062/1503 (70.7)
Bisexual	126/446 (28.3)	354/1503 (23.6)
Heterosexual	30/446 (6.7)	67/1503 (4.5)
Trans	11/446 (2.5)	7/1503 (0.5)
Sexual role		
Active	215/439 (49)	683/1493 (45.8)
Passive	73/439 (16.6)	255/1493 (17.1)
Modern	148/439 (33.7)	545/1493 (36.5)
Other/Don’t know	3/439 (0.7)	10/1493 (0.67)
Type of partner		
Primary	50/443 (11.3)	122/1503 (8.1)
Casual repeated	190/443 (42.9)	661/1503 (44)
Casual one time	183/443 (41.3)	623/1503 (41.5)
Commercial client	5/443 (1.1)	59/1503 (3.9)
Sex worker	0/443 (0)	15/1503 (1)
Other	15/443 (3.4)	23/1503 (1.5)
Age		
Less than 18	3/445 (0.7)	16/1501 (1.1)
18–25	157/445 (35.3)	376/1501 (25.1)
25–35	210/445 (47.2)	716/1501 (47.7)
35–45	65/445 (14.6)	299/1501 (20.0)
45–60	9/445 (2.0)	9/1501 (2.0)
60+	1/445 (0.2)	4/1501 (0.3)
HIV status		
HIV positive	15/447 (3.4)	147/1504 (9.8)
HIV negative	102/447 (22.8)	190/1504 (12.6)
Unknown	330/447 (73.8)	1167/1504 (77.6)
Partner taking PrEP (if HIV neg)?		
Yes	9/102 (8.8)	58/189 (30.7)
No	52/102 (51.0)	72/189 (38.0)
Don’t know	41/102 (40.2)	59/189 (31.0)

### Sensitivity analysis

The sensitivity analysis, using a viral load cutoff of >1000 copies/mL, re-categorized 13 of 665 participants as having “undetectable” viremia. Using this cutoff, 140/665 (21%) were classified as having “detectable” viremia, versus (153/665) 23% in our original analysis. All further results from the sensitivity analysis were not different from those described above. For example, rates of serodiscordant condomless anal intercourse among those with detectable versus detectable viremia did not change from the original analysis. Further, there were no differences in frequencies in the partner characteristics analysis.

## Discussion

Our study is the first to evaluate the frequency of serodiscordant condomless sex and risks for secondary HIV transmission in the sexual networks of MSM in Peru with uncontrolled as well as controlled HIV infection. In this sample of Peruvian MSM living with HIV, almost all reported serodiscordant CAI with multiple partners in the last three months, though individuals with detectable viremia reported serodiscordant condomless anal sex significantly more often than those with an undetectable viral load. Serodiscordant CAI was more common among men who did not know they were living with HIV, and who tended to be younger, to have less education, and to identify as bisexual rather than gay or homosexual. Factors associated with detectable viremia also included younger age and lower level of formal education, and a lower likelihood of prior HIV testing or use of ART. The largest gap in the HIV care cascade was at the first step—diagnosis, with 81.7% of participants previously tested for HIV and 44.0% reporting a prior positive result. This study provides context for the persistently high prevalence of HIV among MSM in Lima, which has been estimated between 10 and 20%.^10^ These findings indicate a critical need for expanded access to HIV testing as a strategy to limit HIV transmission in South America.

The most important finding in our study is the alarmingly high frequency of serodiscordant CAI reported by MSM with detectable HIV viremia. In an observational cohort from 2006, 34% and 45% of men living with HIV in Peru reported insertive and/or receptive CAI with a serodiscordant partner, respectively, with no significant differences noted according to participants’ knowledge of their own HIV serostatus. In a 2014 analysis of Peruvian MSM recently diagnosed with HIV and/or another STI,^6^ CAI occurred with 41.3% of all partners, though it was not specified whether these partnerships were serodiscordant, and that cohort included individuals who both were and were not living with HIV. In the context of our sample, where 94% of all MSM living with HIV reported recent condomless anal sex, men with uncontrolled HIV viremia reported alarmingly high frequencies of both insertive (63%) and receptive (86%) serodiscordant CAI with one or more of their last three partners. Given the expansive sexual networks reported, with a median of 15 (mostly casual and anonymous) recent sexual partners in a three-month period, the elevated viral loads quantified among men with detectable viremia (median: 70,000 copies/mL), and the high frequency of serodiscordant CAI, our data suggest tremendous risks for both HIV acquisition among previously uninfected individuals, and for ongoing secondary HIV transmission within the population.

Among men with both detectable and undetectable viremia, the primary gap in the HIV care cascade was participants’ awareness of their HIV status. Among the 75% of the men in our sample who were aware that they were living with HIV, 94% were on ART and 93% were virally suppressed, nearing the UNAIDS 95-95-95 goals for HIV management and suggesting that, once diagnosed, existing efforts at linkage to and retention in care are effective. However, it has been estimated that only 24% of MSM and TW living with HIV in Peru are aware of their serostatus, with HIV testing representing the largest drop-off in the HIV care continuum among MSM and TW.^2^ Further, sustained engagement with HIV screening in Peru is rare, with as few as 6.2% of MSM and TW in a prior study meeting the Peruvian Ministry of Health’s recommendations for the standard of care for HIV testing every six months.^11^ Our data support and extend these findings, showing that undiagnosed HIV infection is common among men at high risk for acquiring HIV (25% of our sample) and that both high-risk sexual behaviour and uncontrolled HIV-1 viremia are common in this group. Given that men unaware of their HIV-positive status were also more likely to engage in serodiscordant CAI, increasing access to HIV testing is a critical public health problem in Peru.

Barriers to HIV testing included participants’ low self-perceived risk for acquiring HIV, fear of receiving a positive result, and limited access to HIV/STI testing resources.^11^ While the Peruvian Ministry of Health and numerous non-governmental organizations in Lima offer HIV testing free of charge, these services are typically available only at traditional clinic sites during standard operating hours. Several prior studies have assessed the effectiveness of community-based HIV testing, suggesting that these programs could significantly expand the reach of traditional clinic-based testing.^12^, ^13^, ^14^ While the prevalence of HIV infection in these community-based samples (8.2–8.8%) was lower than among individuals tested at HIV/STI clinic venues (16.6–26.3%), the frequency of newly diagnosed HIV infections was high in both settings. More importantly, approximately half of the individuals tested at community sites had never been tested for HIV. The importance of improved access to testing resources was also noted in a study that offered rapid HIV and syphilis testing at parks, bars, and clubs frequented by MSM and TW and reported that over 90% of their participants would test more frequently if testing were routinely offered at these venues.^15^ Similarly, while there is a high degree of acceptability for HIV self-testing among MSM and TW in Peru, the cost and logistics of clinic follow-up are both significant barriers.^16^ Our results highlight the need for increased access to HIV testing and the existing literature suggests that broader access to HIV testing through expanded hours, a wider geographic range of testing sites, and remote testing in community venues, as well as greater availability of resources for secondary HIV prevention, could be key strategies in the local epidemiologic context.

When asked about the HIV status of their last three sexual contacts, participants with detectable viremia did not know the serostatus of 74% of their partners, suggesting that they had not discussed HIV during any of these sexual encounter (This statistic is complicated by the fact that the 25% of participants living with HIV were undiagnosed and would have likely described their HIV status inaccurately). Similarly low rates of knowledge of partner HIV status among MSM in Lima were described by Nagaraj and colleagues, with participants only knowing the serostatus of 14% of their sexual partnerships in the past 3 months and conversations about HIV only occurring within primary or stable partnerships.^17^ Additionally, Konda and colleagues highlighted that less than half of MSM and trans-women in their study population in Lima engaged in serostatus disclosure conversations and even fewer were aware of the serostatus of their partners.^18^ These findings suggest the importance of encouraging routine discussions of HIV with all sexual partners as a strategy to promote knowledge and disclosure of HIV status among men at risk for HIV acquisition and transmission.

There are several limitations to this analysis that need to be considered when interpreting our findings. First, a requirement for participation in the study was that participants report at least one episode of CAI within the last six months, which is likely to have skewed the risk profile of the population and increased the overall frequency of serodiscordant CAI described in our results. Additionally, participants were asked to self-report the number of partners with whom they had CAI, which may result in an underestimation of the true number of events due to social desirability and recall bias. However, we attempted to minimize the potential for bias by using CASI surveys and emphasizing participant confidentiality and privacy during sexual network interviews. Serodiscordant CAI was based on respondents’ reports, which also may not be accurate. To address this concern, we assumed that all unknown-status partners were serodiscordant, to give the most conservative estimate. Regardless of the true serostatus of these partners, status disclosure was uncommon, which itself has important ramifications for HIV transmission. Importantly, we used a cut off of >200 copies/mL to define detectable viremia, which is based on prospective data from the PARTNER trial^7^ and is the current standard of care in clinical practice. However, we conducted a sensitivity analysis using a cut-off of >1000 copies/mL based on results of a recent systematic review suggesting there is low likelihood of transmission under this cut-off.^19^ Our results did not change from the original analysis, speaking to their robustness. Finally, the COVID-19 pandemic had a large impact on access to testing during Peru’s lockdowns,^20^ and may have contributed to the observed gap in the diagnosis step of the HIV care cascade. However, as noted above, the frequency of HIV testing and undiagnosed HIV infection in our sample is consistent with pre-COVID studies conducted among MSM in Peru and a primary driver of Peru’s contemporary HIV epidemic.

### Conclusions

In our population of MSM living with HIV in Lima, Peru, there was a high prevalence of uncontrolled HIV-1 infection and serodiscordant CAI, suggesting a basis for Peru’s persistent HIV epidemic among MSM and TW. The alarming frequency of serodiscordant CAI among men with detectable viremia is an urgent public health issue and must be rapidly addressed. Consistent with prior literature, we found the main gap in HIV care cascade in this population at the level of diagnosis. Innovative outreach strategies to increase testing should be funded on a national scale to close this care gap. Strategies should be diverse and include resources to expand the hours and locations of testing options to include nights, weekends, and venues frequented by MSM, TW, and sex workers. In addition to promoting HIV/STI testing, disclosure and discussion of HIV status should be encouraged through programs to reduce HIV-related stigma and to support partner notification following a new HIV or STI diagnosis. Together, increased availability of HIV/STI testing along with the cultural normalization of HIV serostatus discussions among MSM and TW in Peru would dramatically reduce the frequency of secondary HIV transmission within these networks.

## Contributors

CH is the primary author and contributed to the conceptualization, data curation, formal analysis, investigation, and methodology of the project, and wrote the original draft of the manuscript.

ES contributed to the conceptualization, data curation, formal analysis, methodology, software, and supervision of the project, and reviewed and edited the manuscript.

JG contributed to the data curation, investigation, and administration of the project, and reviewed and edited the manuscript.

JL contributed to the conceptualization, methodology, and supervision of this project, and reviewed and edited the manuscript.

RC contributed to the conceptualization, investigation, project administration, resources, and supervision of this project, and reviewed and edited the manuscript.

CB contributed to the conceptualization, data curation, formal analysis, methodology, and supervision of this project, and reviewed and edited the manuscript.

JC contributed to the conceptualization, data curation, formal analysis, methodology, funding acquisition, administration, and supervision of this project, and reviewed and edited the manuscript.

CH, ES, CB, and JC had access to and verified the raw data.

All authors agreed with the decision to submit the manuscript.

## Data sharing statement

Due to participant confidentiality and ethical considerations, individual-level data cannot be made publicly available. In order to obtain access to the data, approval from the UCLA Office of the Human Research Protection Program is required. Authors that meet the criteria for access for confidential data may submit requests by email to Dr. Jesse Clark (jlclark@mednet.ucla.edu).

## Declaration of interests

None of the authors on this paper have financial or other conflicts of interest to disclose.

## Appendix ASupplementary data

Translated abstract spanish_disclaimer
