# Supplementary material for: Sexual network characteristics, condomless anal intercourse, and the HIV care cascade among MSM living with controlled versus uncontrolled HIV infection in Lima, Peru: a population-based cross-sectional analysis
Source: Lancet Reg Health Am. 2024 Mar 27;32:100722. doi: 10.1016/j.lana.2024.100722 (PMC11019357; doi:10.1016/j.lana.2024.100722)
Supplement: Translated abstract spanish_disclaimer [file mmc1.docx]

***Editorial Disclaimer:*** *This translation in Spanish was submitted by the authors and we reproduce it as supplied. It has not been peer-reviewed. Our editorial processes have only been applied to the original abstract in English, which should serve as a reference for this manuscript.*

**Resumen**

**Contexto:**

A pesar de las altas tasas de transmisión del VIH entre hombres que tienen sexo con hombres (HSH) en Lima, Perú, existen datos limitados sobre las características de las redes sexuales o de los factores de riesgo de transmisión secundaria del VIH entre los HSH con infección VIH no controlada. Presentamos la frecuencia de relaciones sexuales anales (RSA) serodiscordantes y sin condón y las características de las redes sexuales asociadas entre HSH con viremia detectable y los comparamos con aquellos que tienen viremia indetectable.

**Métodos:**

Este análisis transversal incluye a HSH que dieron positivo en la prueba de detección del VIH-1 durante la fase de elegibilidad para un ensayo clínico de manejo de parejas y control de ITS (junio de 2022-enero de 2023). A los participantes tamizados se les realizaron pruebas de VIH, gonorrea, clamidia y sífilis, y completaron cuestionarios sobre sus características demográficas, identidad y comportamiento sexual, estructuras de redes sexuales, y vinculación a programas de atención VIH.

**Resultados:**

De los 665 HSH, 153 (23%) tenían viremia detectable (>200 copias/mL). El 75% (499/662) de los hombres que viven con el VIH fueron diagnosticados previamente, el 94% (n = 469/499) informaron que estaban en tratamiento antirretroviral y el 93% (n = 436/469) tenían supresión viral. El 96% (n = 147/153) de los hombres con viremia detectable informaron RSA sin condón y serodiscordante con al menos una de sus últimas tres parejas sexuales, y el 74% (n = 106/144) informaron lo mismo con sus tres parejas recientes. Por el contrario, el 62% (n=302/489) de los hombres con viremia indetectable informaron RSA sin condón y serodiscordante con sus tres últimas parejas (p <0,01).

**Interpretación:**

El 23% de los hombres que viven con el VIH en el Perú tenían viremia detectable, de los cuales casi todos (96%) informaron RSA sin condón y serodiscordante reciente. La principal brecha en la cascada de atención del VIH radica en el conocimiento del estado serológico del VIH, lo que sugiere que un mejor acceso a las pruebas del VIH podría ser una estrategia de prevención clave en Perú.

**Financiamiento:**

El financiamiento de este estudio provino de las subvenciones R01 MH118973 (PI: Clark) y R25 MH087222 (PI: Clark) de los NIH/NIMH.
